# Supplementary material for: Neutralizing Antibodies in COVID-19 Serum from Tatarstan, Russia
Source: Int J Mol Sci. 2023 Jun 15;24(12):10181. doi: 10.3390/ijms241210181 (PMC10299584; doi:10.3390/ijms241210181)
Supplement: Supplementary file 1 [file ijms-24-10181-s001.zip › ijms-2413122-supplementary.pdf]

Supplementary Table S1. Inhibition rate of neutralizing antibodies in COVID-19 patients.

Inhibition rate of neutralizing antibodies in Fatal vs Non-fatal COVID-19 (81 patients)			
	Fatal	Non-fatal	P value
Omicron Neutralizing antibodies, %	16.30±30.12	28.15±37.34	0.28
Wuhan Neutralizing antibodies, %	35.20±48.20	43.53±41.50	0.56
**Inhibition rate of neutralizing antibodies in severe COVID-19 (27 patients)**			
	Fatal	Non-fatal	P value
Omicron Neutralizing antibodies, %	17.47±30.90	6.37±22.98	0.22
Wuhan Neutralizing antibodies, %	35.20±48.20	17.93±40.10	0.70
**Inhibition rate of neutralizing antibodies in all patients receiving with/without olokizumab (81 patients)**			
	-	+	P value
Omicron Neutralizing antibodies, %	31.46±39.00	20.04±32.46	0.21
Wuhan Neutralizing antibodies, %	49.58±41.64	33.49±41.07	0.25
**Inhibition rate of neutralizing antibodies in COVID-19 patients in patients with different ethnicity (81 patients)**			
	Russian	Tatar	P value
Omicron Neutralizing antibodies, %	26.13±36.80	25.84±36.23	0.97
Wuhan Neutralizing antibodies, %	40.11±42.50	44.78±41.83	0.97

N – number of patients; % - percent from patients included into the group.

Supplementary Table S2. Frequency of neutralizing antibodies detection in COVID-19 patients with and without olokizumab treatment.

**All COVID-19 samples (81 patients)**						
	Omicron Neutralizing antibodies, n (%)			Wuhan Neutralizing antibodies, n (%)		
olokizumab	presence	absence	p value (Fisher exact test)	presence	absence	p value
-	18 (42%)	25 (58%)	0.487	18 (64%)	10 (36%)	0.257
+	11 (28%)	28 (72%)		8 (38%)	13 (6%)	
**Mild COVID-19 (24 patients)**						
	Omicron Neutralizing antibodies, n (%)			Wuhan Neutralizing antibodies, n (%)		
olokizumab	presence	absence	p value (Fisher exact test)	presence	absence	p value
-	5 (24%)	16 (76%)	1	5 (56%)	4 (44%)	1
+	1 (33%)	2 (67%)		1 (100%)	0 (0%)	
**Moderate COVID-19 (30 patients)**						
	Omicron Neutralizing antibodies, n (%)			Wuhan Neutralizing antibodies, n (%)		
olokizumab	presence	absence	p value (Fisher exact test)	presence	absence	p value
-	11 (65%)	6 (35%)	0.711	11 (65%)	6 (35%)	0.471
+	7 (54%)	6 (46%)		6 (50%)	6 (50%)	
**Severe COVID-19 (27 patients)**						
	Omicron Neutralizing antibodies, n (%)			Wuhan Neutralizing antibodies, n (%)		
olokizumab	presence	absence	p value (Fisher exact test)	presence	absence	p value
-	1 (25%)	3 (75%)	1	1 (50%)	1 (50%)	1
+	4 (17%)	19 (83%)		2 (25%)	6 (75%)	

N – number of patients; % - percent from patients in each group.
P values were calculated using exact Fisher test with BH correction.

Supplementary Table S3. Frequency of COVID-19 diagnosis in patients depending on ethnicity

	Russian	Tatar	P value
Mild COVID-19 n (%)	10 (42%)	14 (58%)	Mild vs. Moderate: 0.59
Moderate COVID-19 n (%)	15 (50%)	15 (50%)	Mild vs. Severe: 0.39
Severe COVID-19 n (%)	8 (30%)	19 (70%)	Moderate vs. Severe: 0.18

N – number of patients; % - percent from all 81 patients included into this study;
P values were calculated using exact Fisher test with BH correction.

Supplementary Table S4. Frequency of anti-SARS-CoV-2 neutralizing antibodies detection in COVID-19 patients by ethnicity

**All COVID-19 samples (81 patients)**						
	Omicron Neutralizing antibodies, n (%)			Wuhan Neutralizing antibodies, n (%)		
Ethnicity	presence	absence	p value	presence	absence	p value
Russian	12 (36%)	21 (64%)	1	11 (50%)	11 (50%)	0.581
Tatar	17 (35%)	31 (65%)		17 (59%)	12 (41%)	
**Mild COVID-19 (24 patients)**						
	Omicron Neutralizing antibodies, n (%)			Wuhan Neutralizing antibodies, n (%)		
Ethnicity	presence	absence	p value	presence	absence	p value
Russian	3 (30%)	7 (70%)	0.665	3 (50%)	3 (50%)	1
Tatar	3 (21%)	11 (79%)		3 (50%)	3 (50%)	
**Moderate COVID-19 (30 patients)**						
	Omicron Neutralizing antibodies, n (%)			Wuhan Neutralizing antibodies, n (%)		
Ethnicity	presence	absence	p value	presence	absence	p value

Russian	7 (47%)	8 (53%)	0.665	7 (50%)	7 (50%)	0.462
Tatar	11 (73%)	4 (27%)		10 (67%)	5 (33%)	
**Severe COVID-19 (27 patients)**						
	Omicron Neutralizing antibodies, n (%)			Wuhan Neutralizing antibodies, n (%)		
Ethnicity	presence	absence	p value	presence	absence	p value
Russian	2 (25%)	6 (75%)	0.616	1 (25%)	3 (75%)	1
Tatar	3 (16%)	16 (84%)		2 (33%)	4 (67%)	

N – number of patients; % - percent from patients in each group;
P values were calculated using exact Fisher test with BH correction.
